# Wireless Electrical Stimulators and Sensors Network for Closed Loop Control in Rehabilitation

**DOI:** 10.3389/fnins.2020.00117

**Published:** 2020-02-19

**Authors:** David Andreu, Benoît Sijobert, Mickael Toussaint, Charles Fattal, Christine Azevedo-Coste, David Guiraud

**Affiliations:** ^1^CAMIN, INRIA, University of Montpellier, CNRS, Montpellier, France; ^2^Vivaltis, Montpellier, France; ^3^CRF La Châtaigneraie, Menucourt, France

**Keywords:** functional electrical stimulation, neuroprosthesis, sensory-motor deficiencies, motor rehabilitation, wireless FES architecture

## Abstract

This paper presents a wireless distributed Functional Electrical Stimulation (FES) architecture. It is based on a set of, potentially heterogeneous, distributed stimulation and measurement units managed by a wearable controller. Through a proof-of-concept application, the characterization of the wireless network performances was assessed to check the adequacy of this solution with open-loop and closed-loop control requirements. We show the guaranteed time performances over the network through the control of quadriceps and hamstrings stimulation parameters based on the monitoring of the knee joint angle. Our solution intends to be a tool for researchers and therapists to develop closed-loop control algorithms and strategies for rehabilitation, allowing the design of wearable systems for a daily use context.

## 1. Introduction

Electrical Stimulation (ES) induces Action Potentials (AP) by depolarizing the membrane of the targeted cells in particular axons or muscle fibers at the motor point. Since the 1950's, ES has been successfully used in a growing set of applications linked to motor and sensory impairments. Attempts to use ES have been made in movement rehabilitation, such as drop foot syndrome correction for post-stroke hemiplegic patients (Liberson et al., 1961) and more complex movements or functions for patients with a spinal cord injury (Kralj and Bajd, 1989; Davis et al., 1997; Kobetic et al., 1997, 1999; Rijkhoff, 2004; Guiraud et al., 2006a,b). In Smith et al. (1998), the functional results are substantial including, for instance, recovery of the grasp function for quadriplegic patients, who might then be able to grab and hold objects, eat, and even, in the best cases, write with a pen. Although not optimal, Functional ES (FES) systems remain the only way to date to restore paralyzed muscle's contraction so they are valuable tools for acute clinical rehabilitation. Besides, recently, researches for movement restoration through muscle's activation of the lower limb in particular, regained interest through new surgical approaches and stimulation targets (Possover et al., 2010; Harkema et al., 2011; Angeli et al., 2018; Wagner et al., 2018). In these papers, the authors described the abilities of the spinal cord to generate useful muscle activation that may provide standing and even walking patterns. However, as commonly stated in literature, available stimulators, both implanted and external, remain too limited to explore widely all the possibilities that these techniques could provide. Among these limitations, functional movements controlled in a closed loop way are still unused, except in focused research protocols, although it is known that the human nervous system is controlling movement through complex multilevel closed loops. Indeed, an efficient functional movement would need for closed loop control (balance control, fatigue compensation) or optimized synthesized patterns (sit to stand movement, grasping). It means that several stimulation points and sensors have to be placed on the body. It leads to complex donning and doffing in particular due to classical wired links. Thus, wireless systems appear to be a neat solution, *a fortiori* wearable to meet the need for mobility, however it leads to issues when safety and guaranteed performances to achieve closed loop control are mandatory.

The first network based FES system available was the BION (Loeb et al., 2001). The implantable, thus invasive, technology faced the difficulty to power the system through external inductive antennas over wide areas of the body, even with its rechargeable version. Moreover, closed loop control, as far as we know, was never used on such network finally dedicated to acute rehabilitation. Some external FES stimulators already use wireless technology, mainly to allow portability (Broderick et al., 2008; Chae et al., 2008), i.e., stimulators that can be worn by the patient without being physically connected to a computer. Some of these stimulators are standalone units, meaning that one of the available programs can directly be selected or parameterized on the stimulator itself. Some examples of existing products are the Compex Wireless from Compex, the NESS L300 from Bioness (Hausdorff and Ring, 2008; Dunning et al., 2009; Laufer et al., 2009), the WalkAid from Innovative Neurotronics (Weber et al., 2005). These systems are designed to carry out a unique thus specific FES-application. Even if these stimulators can be used in different FES therapies, it is still impossible to use the technology for multi-site FES applications despite their 4-channel outputs for some. Indeed, to treat different functional deficiencies eventually simultaneously, it is necessary to coordinate stimulation and acquisition on distributed sites on the human body. To achieve this task, it is necessary to connect and coordinate stimulators via the network, and today, very few external wireless FES stimulators attempt to do so: Jovicic et al. propose a prototype but discusses mainly the problem of transmission and relay between units and a host computer (Jovicic et al., 2012). An efficient routing protocol has been proposed to face frame losses (due to signals' attenuation) when communicating with the mandatory remote computer since the system is not fully wearable, and thus not adapted to daily life context.

To guarantee safety and performances through wireless link, the key issue is the Medium Access Control (MAC) protocol. Prototypes of networked implantable neuroprothesis for which we already designed stimulation units (Andreu et al., 2009), present another application with close MAC protocol design but not used on a wireless medium. The purpose of the paper is to detail the adequacy of our open, potentially heterogeneous, wireless architecture—hardware, software, and protocol—with closed loop control requirements over a distributed FES system.

The paper is organized as follows: the distributed architecture is described, then wireless network properties are related to the closed loop control requirements, quantitative results show the real performances of the system followed by a relevant illustrative clinical application.

## 2. Materials and Methods

### 2.1. Distributed FES Architecture

The underlying principle of distributed architectures is to decentralize parts of the processing on a set of physical distributed units (DU). Activities of these entities are coordinated at higher levels of the architecture to provide complex functionalities. According to this principle, we designed an external wireless FES architecture based on:

- Distributed Stimulation Units (DSU): a DSU executes locally the stimulation profile and thus generates the stimulus.
- Distributed Measurement Units (DMU): currently, DMU can acquire EMG or physical data such as angles (goniometers), 3D accelerations, or inertial sensors. Thus, a DMU locally performs the acquisition of the signals and processes data, such as digital filtering, envelope computation, or threshold detection.
- Control Unit (CU): the controller is in charge of coordinating activities of DSUs and DMUs to offer high level functionalities according to the running FES application. CU configures, coordinates and schedules all DUs. CU remotely modulates relevant parameters of DSUs and collects processed data from DMUs for closed loop control purposes. Finally, CU supervises and controls the network Quality of Service (QoS).
  CU can be used in a standalone mode—within a "homogeneous" architecture implying only the CU and a set of DSUs and DMUs—or as a gateway between this networked FES system and a wearable controller (or a computer) that then ensures the closed loop control and the connections (wired or wireless ones) with other types of sensors, leading to a "heterogeneous" architecture involving multiple networks (e.g., sensor networks). This allows interfacing with any kind of sensors while keeping the most critical part, i.e., the DSU, unchanged. The 2 types of architectures are illustrated through the paper.

### 2.2. Wireless Communication Link

Communication is a critical issue as it directly has an impact on the performances, the reliability and the safety of the system. Indeed, compared to wired or centralized systems, a wireless system over has to face: (i) avoidance of collision, (ii) optimization of bandwidth occupation, (iii) determinism, (iv) bounded time latencies for robust control, (v) safety against frame losses.

Wifi technology (802.11) cannot be used since the CSMA/CA method does not offer a deterministic MAC, and its PCF (Point Coordination Function) mode is not efficient, even not always implemented. Bluetooth solution (IEEE 802.15.1) has an important drawback considering the need for network synchronization delays in scatter-nets of multiple piconets (small networks up to 8 slaves). ZigBee technology (IEEE 802.15.4) provides deterministic medium access through guaranteed time slots within the contention free period on beacon-enabled network. It is moreover efficient in its use of power and able to support a network with thousands of devices thanks to a cluster tree or mesh network's topology. However, it is more adequate for communication between devices and services dedicated to remote monitoring than for real-time FES closed-loop control. Indeed, Zigbee is used for applications where the latency of transmission is not critical (transmit GTS and/or receive GTS nodes' request and coordinator GTSs management impacts efficiency, and the routing layer as well). Its software architecture, protocol stack and services, remains complex. However, it relies on a low-power digital radio based on the IEEE 802.15.4 standard which is efficient in terms of receiver sensitivity, link quality indication, transmit power adjustment. We thus use only the physical layer of this technology.

We developed a communication solution based on a 3-layer protocol stack (reduced OSI model): physical, MAC and application layers. The physical layer (2.4 GHz RF link, IEEE 802.15.4) has a bit-rate of 250 Kbs^−1^. The less occupied channel is detected to limit interference and then enhance the QoS of the wireless link; reception and emission power are measured and adapted to optimize power consumption and link reliability. Besides, this physical layer can be changed if needed, according to new communication standard, enhanced technology or local country rules *a fortiori* in medical-context applications (Baker and Hoglund, 2008; Panescu, 2008). The application layer supports configuration, programming and remote operating of the DUs and allows for a very flexible evolution toward new type of DU without changing physical and MAC layers.

The main issue remains the MAC aspects. Due to limitations of existing solutions, an original MAC protocol was designed in order to minimize the risk of collisions between frames and to optimize medium sharing, both for efficiency and reactivity purposes. We designed the Sliding Time Interval Medium Access Protocol (STIMAP). It ensures that only one DU communicates over the network at a given time while optimizing the bandwidth use through a smart adjustment of time slots duration (Godary et al., 2007; Godary-Dejean and Andreu, 2013). STIMAP is based on the Master/Slaves model—the CU being the master and other DUs being slaves—and dynamic TDMA (Time Division Multiple Access, see Appendix). STIMAP offers unicast, multicast, and broadcast addressing. Multicast addressing allows grouping of DUs. A DU can be member of up to 8 groups. Address space allows for defining 64 groups and 64 DUs over one network; this being, the maximum number of units that can be involved in this wireless architecture depends both on the time constraints of the application and on the number of sensors (since it is the exchange of sensor data that consumes the most bandwidth). The main properties of this original MAC protocol are:

- Multicast provides: (i) simultaneous addressing of several DUs with a unique frame minimizing medium occupancy (ii) network level synchronization (beacons like) (Godary-Dejean and Andreu, 2013).
- Adjustable and optimized time-slot allocation ensures a better trade-off between reactivity / answer to a request from a DU, and time slot occupancy through dynamic unused time-slot recovery.

### 2.3. Hardware and Software Architectures of Units

**Figure 1:**
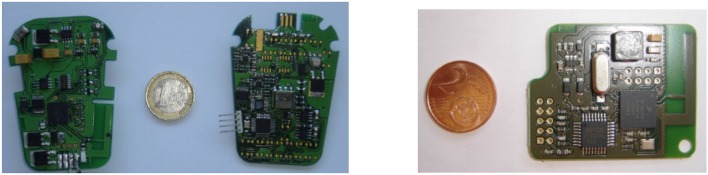
**(Left)** A distributed stimulation unit is composed of a communication-dedicated board and a stimulation one. **(Right)** The controller board manages the wireless network and the real-time application.

A CE-marked external FES system based on our distributed wireless architecture was developed in collaboration with Vivaltis Company (Montpellier, France). The CU board can be connected to a wearable controller or a computer via an USB link, allowing the practitioner to configure and program the complete system through application specific GUIs. The CU, worn by the patient, ensures all communications with DUs and provides for the scheduling of DU activities including closed-loop control in standalone mode (i.e., homogeneous architecture). DU relies on a 2-board based architecture: a generic board embedding the communication protocol stack and a specific board composed of digital and analog electronics adapted to stimulation or acquisition (Figure 1, left). Unit's volume and weight are respectively 80*55*30 mm^3^ and 98 g.

#### 2.3.1. Distributed Stimulation Unit

**Figure 2:**
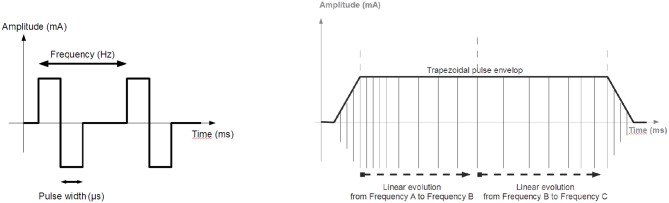
Stimulation: **(Left)** Biphasic pulses stimulation profile generated by the distributed stimulation unit. **(Right)** Envelope of the pulse train.

The stimulation unit is a regulated-current 2-channel stimulator, able to sequentially deliver a stimulus on each channel. The features are: maximal current 100 mA, 0.1 mA step on a maximum load of 1 *k*Ω, stimulation frequency 1 Hz to 1 kHz, pulse-width 50 μ*s* min., 1 μ*s* step, and electrical polarity can be configured. All parameters are dynamically and remotely adjustable (Figure 2).

#### 2.3.2. Distributed Measurement Unit

Specific boards can be developed to interface with various types of sensors such as accelerometers or Inertial Motion Units (IMU). The 2-channel EMG unit (Vivaltis, France) can alternately sample two input signals. The features are: a bandwidth from 10 Hz to 1 kHz, sampling frequency of 2.5 kHz per channel, 3 programmable input ranges (80, 200, and 400 μ*V*), and a digital resolution of 12 bits. The DMU can numerically rectify and filter EMG with a programmable cut-off frequency to get the envelope thus limiting the necessary bandwidth on the medium by using under-sampling. A second DMU was designed with a 2D goniometer (Biometrics). This DMU samples both angles at 25 Hz with a 12-bit resolution.

#### 2.3.3. Hardware and Software Implementations

**Figure 3:**
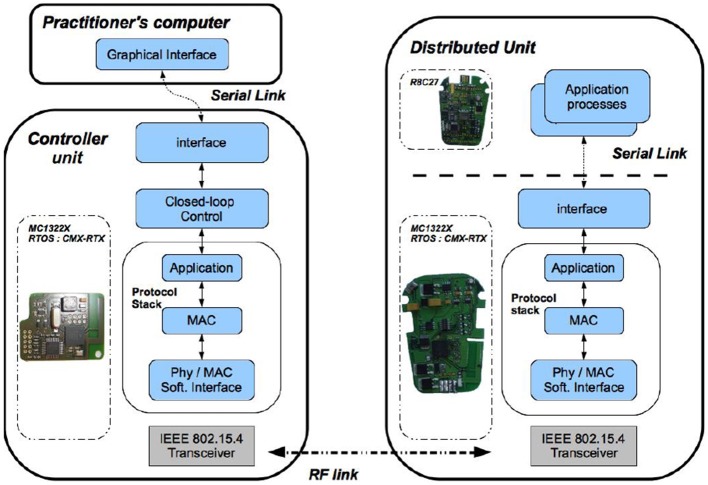
Software modules deployment **(Left)** On the control unit board, embedded real-time software architecture manages both the wireless communication and the closed loop control. **(Right)** On the distributed unit board, the embedded micro-controller runs the real-time communication stack and controls the stimulation process.

For DSU and DMU, the software architecture is based on the same set of tasks deployed on two microprocessors (Figure 3), for the generic (ARM7 of Freescale MC1322X, with the RTOS CMX-RTX) and specific boards (Renesas R8C27).

The CU is based on a microprocessor board (ARM7 of Freescale MC1322X), (Figure 1). Its software application is multitask (Figure 3), running on a real-time operating system (RTOS CMX-RTX).

#### 2.3.4. Safety Issues

There are 2 levels of safety; the first one concerns stimulation generation. On the stimulation board, a 10-bit ADC measures the effective output current on a serial shunt resistor (2 Ω). Open-circuit or saturated output can be detected (due for instance to high impedance of the electrode). This current is checked on the DSU and limited by software depending on the application. It ensures that the DSU cannot deliver more current than it is supposed to. It is all the more important on wireless systems, that transmission failures may occur more easily than on wired systems.

The second level of safety deals with the wireless link issue. Since the DU is an autonomous unit being remotely controlled, the CU periodically checks if the DU is still communicating by means of presence test requests (section 3). Absence of DU acknowledgment can have several causes: communication (unreachable node), software application (DU locally stopped), and insufficient power. If the DU does not acknowledge a defined number of successive presence tests then the CU notifies the user, stops the application and goes in a predefined safe mode. On the DU side, the same safety check is performed: if the DU does not receive presence test requests for 3 s, then it stops its activity and shuts down itself in a predefined safe mode.

#### 2.3.5. Power Issues

Each DU is powered by a rechargeable battery (3.7 *Wh*, 1,000 *mAh*). Measured power consumption of a DSU, including communication, is 2 W (260 *mA*, 7.4 *V*) with stimulation parameters being: 20 *mA* amplitude, 400 μ*s* pulse width, and 100 *Hz* frequency on both channels. Considering the battery capacity, the autonomy is about 4 h. For standard stimulation with parameters set to 20 mA amplitude, 200 μ*s* width, and 50 *Hz* frequency, the autonomy would be more than 11 h.

## 3. Results

Advanced rehabilitation protocols would benefit from closed loop control but it requires determinism, time performances and safety. The results show the capabilities of our architecture and its devices to fulfill these requirements. We first defined a proof-of-concept experimental setup to characterize the performance of the system, based on a homogeneous architecture (i.e., implying only CU, DSUs, and DMUs). Then, this open wireless architecture has been extended to a heterogeneous version with a wearable controller and several wired and wireless sensors, and used in the context of a clinical protocol: FES-based control of knee joint to reduce stance phase asymmetry in post-stroke gait.

### 3.1. Proof-of-Concept Experimental Tests

**Figure 4:**
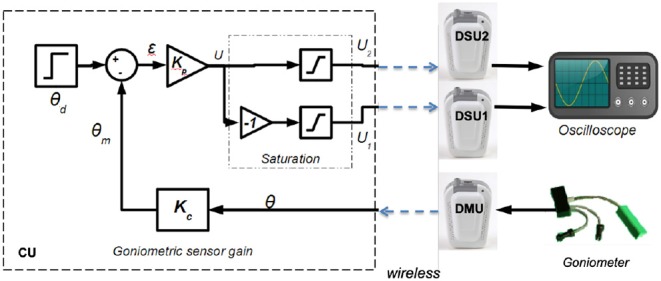
Multiple muscle contraction control scheme based on proportional gain using 3 distributed units and 1 control unit.

We defined an experimental setup that includes a goniometer—1 DMU—for measuring a joint angle. It controls the contraction of 2 antagonist muscles acting on this joint—2 DSU. We observed the system outputs (both stimulation patterns) on a dummy load (Figure 4). This control scheme is not evaluated *per se*, but the whole system is provided to assess the following features: (i) wireless link properties in particular linked to the original MAC protocol we designed, (ii) real-time performances in particular timing and synchronization allowed by our original architecture associated with MAC protocol.

The control scheme is based on proportional gain while stimulating either the agonist or antagonist muscle according to the sign of tracking error (Figure 4).

Besides, in a closed loop control scheme, as muscles time response is around 100 ms (Vette et al., 2008) and stimulation period around 40 ms, the sampling period of the command is set to 40 ms without any loss of controllability. The proportional gains are *Kp* = 0.5 and *Kc* = 1 and include voltage to angle conversion. There is a saturation between the proportional error output and the actual stimulation intensity acting on muscles to avoid over stimulation of muscles. These maximum amplitudes are set for each muscle *a priori*, within safe limits.

#### 3.1.1. Wireless Link Performances

In this characterization, the CU is responsible for dating events and collecting transmission parameters: time stamping requests and corresponding acknowledgments to calculate the round-trip time (RTT) between CU and DU communications, collecting the link quality indicator (LQI, measuring the signal quality level of the frame reception) and determination of frame losses (number of non-received acknowledgments) among 5,000 dummy frames exchange (100 bytes long).

##### 3.1.1.1. Performances of the physical layer

Frame losses are due to collisions of frames or perturbations which can occur when other wireless technologies are used in the same environment around the 2.4 GHz RF band as local area networks (Wi-Fi IEEE802.11), wireless personal area networks (Bluetooth IEEE802.15.1) and Zigbee (IEEE802.15.4).

At power on, the CU and DUs are configured with a default channel. After having determined the least occupied channel from the 16 available ones, the CU indicates to all DUs the channel selected to communicate safely, i.e., with the least disturbances from other wireless technologies.

Moreover, as soon as a frame is received the physical coupler supplies a measure of the received RF signal power. This LQI is between –15 dBm for a good reception quality to –100 dBm for a bad reception quality. The CU observes the LQI evolution to detect any potential impact of the environment on the received signal power, since it can induce frame losses. In the worst case, CU connected to a computer and DUs worn by the patient, LQI is about –75 dBm in case of body opposition: with more than 10,000 exchanges composed of 2 frames each, only 3 DMU and 7 DSU frames were lost in such case. This loss rate can easily be managed by the CU / DU safety procedures without any functional impact, and no frame loss occurs when both CU and DU are worn by the patient.

**Figure 5:**
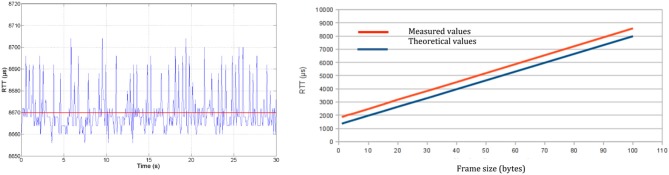
Round-trip time at physical layer (*RTT*_*PHY*_): **(Left)** Evolution with time of 100-byte frame transmission—mean *RTT*_*PHY*_ of 8,670 μ*s* with a standard deviation of 9 μ*s*. **(Right)** Evolution of the transmission time according to frame size.

As physical transmission impacts closed-loop control design, we perform RTT measurements at the physical layer (*RTT*_*PHY*_), i.e., directly from the software interface of the physical layer that pilots the radio transceiver (Figure 5, left). Experiments show that transmission durations increase linearly with the number of transmitted bytes (Figure 5, right). Thus, a minimum *RTT*_*PHY*_ can be estimated knowing the number of bytes exchanged, allowing to set some closed-loop control parameters. Moreover, *RTT*_*PHY*_ is needed to set the time-slot duration of the STIMAP protocol (see Appendix) and set the timeout for monitoring CU to DUs communications.

##### 3.1.1.2. Performances of the MAC layer

As mentioned in section 2, the CU is in charge of configuring groups of DUs (group size, priority, time-slot, etc.). *RTT*_*MAC*_ are measured at MAC layer within the protocol stack, meaning that both MAC software module and physical layer interface software module are taken into account. First, individual (unicast) communications are evaluated for requests like node configuration (MAC parameters setting) and test of presence (similar to the ping protocol). The smallest *RTT*_*MAC*_ is for a test of presence request (2.850 ms) and the highest is for the node configuration (3.080 ms). Then, group based communications are evaluated to verify time-slot positioning at MAC layer since it is essential to ensure the absence of collision (no time-slot overlap) in this context of real-time control: such deterministic MAC protocol ensures an optimized balance between reactivity and time slot occupation (Godary et al., 2007; Andreu et al., 2009).

##### 3.1.1.3. Performances at application layer

The application layer of a DU, executed on the communication board, is in charge of extracting and decoding data from application request sent by the CU. However, the operating mode is not always the same depending on the application, i.e., stimulation or acquisition. Let's first consider the stimulation case. The CU initiated a stimulation sequence by a configuration request sent to the DSU, defining default stimulation parameters as: pulse pattern (Figure 2, left), pulse amplitude, pulse width and frequency. Then the stimulation sequence is enabled by a start request sent to the DSU. Locally the DSU executes an amplitude or a frequency modulation (Figure 2, right) without any other communication. However, during the stimulation sequence execution, the CU can remotely modify the frequency, the pulse width and the current amplitude. So *RTT*_*APP*_ of each operation (Table 1) is evaluated since these values must be taken into account in the design of the FES closed-loop control scheme.

**Table 1:** Round-trip time at application layer (*RTT*_*APP*_) for stimulation operations.

**Operation**	***Mean RTT*_*APP*_ (ms)**	**Std deviation (ms)**
Configuration	12.19	0.016
Stimulation start	5.92	0.011
Amplitude modulation	6.178	0.018
Pulse width modulation	6.012	0.015
Stimulation stop	5.99	0.009

Regarding acquisition operation, the DMU dedicated to goniometers is able to store in a circular buffer up to the last 150 data samples. The measured mean *RTT*_*APP*_ for one data sample gathering is 3.023 ms with a standard deviation of 15 μ*s*. For goniometers the sampling period is equal to the command sampling period, i.e., 40 ms. For EMG, if we want to transmit the envelope, the sampled signal is filtered on the specific board (cut-off frequency is set to 5 Hz) avoiding raw data transmission. This drastically decreases the data rate transfer over the network down to a sample each 40 ms requiring a useful data throughput of 300 *bs*^−1^ instead of 30 *kbs*^−1^. The difference between performances of stimulation vs. acquisition requests comes from the local operating modes: regarding acquisition data are periodically transmitted to the CU (data acquisition and data gathering are independent processes), while concerning stimulation the modulation is effective when applied by the DSU stimulation board (and not only once the message has been received by the DSU).

#### 3.1.2. Distributed Stimulation Synchronization Performances

To evaluate the accuracy of synchronization process at the network level, we estimate the time lag between different stimulations induced when simultaneously starting two DSUs. They may be placed to different sites actuating joints simultaneously by a coordinated stimulation of a 2 pairs of agonist and antagonist muscles (wrist for instance). Tests were performed using two DSUs configured with the same stimulation profile and associated to the same DSU group. Then, they have been started through a group-addressed request. The measured time lag between the 2 first pulses generated by each DSU is 16 μ*s*. This impact is negligible compared to the closed-loop control period and the muscle bandwidth (section 3.1) and demonstrate the accuracy of the protocol stack timing for advanced stimulation synchronization over the network.

### 3.2. Results With FES-Based Knee Joint Control

We developed a closed-loop architecture for FES-based control of knee joint to reduce stance phase asymmetry in post-stroke gait (Sijobert, 2018). We do not present the clinical results but the closed loop performances of the system. However, in few words, the clinical rationale was that the process of gait recovery in patients with severe post-stroke hemiplegia does not only require the control of the foot dorsiflexion but also that of the knee joint. Indeed, it greatly impacts the entire gait cycle and notably the support phase quality. Usual disorders are knee hyperextension during the stance phase (genu recurvatum) and flexed knees (crouch gait). FES is an effective alternative to fixed orthoses to produce appropriately timed knee flexion or extension.

The designed closed loop system aimed at ensuring a safe knee joint lock to allow patients to rely on their paretic leg and transfer their weight onto it during the stance phase. Quadriceps and hamstring are electrically stimulated to ensure that knee extension and flexion are restricted to a safe and physiological range of motion, depending on the gait phase. To do so, a set of sensors is used to detect the gait phases and knee angle evolution, according to which stimulation levels are modulated.

The corresponding protocol was approved by a national ethical committee and participants have signed an informed consent. 11 participants have been included.

#### 3.2.1. Knee Joint Control Experimental Setup

**Figure 6:**
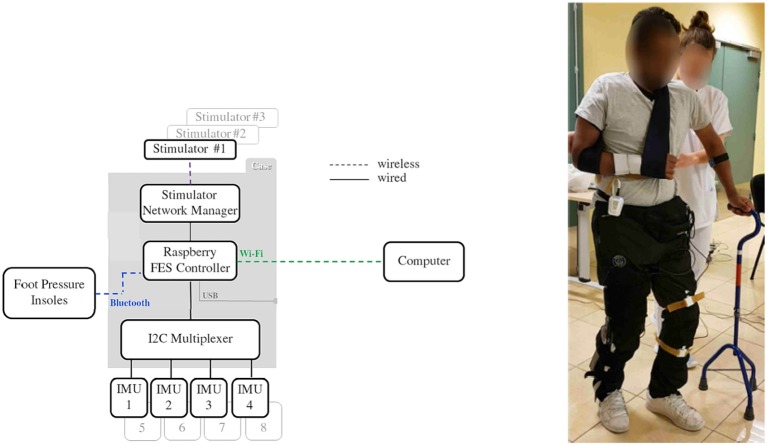
Architecture used for knee control. Subject equipped with inertial measurement unit sensors, stimulator and controller (© Inria / Photo L. Jacq).

The heterogeneous architecture developed for this FES based knee joint control protocol is described in Figure 6. Wireless and wired sensors feed the Raspberry (wearable controller) running a porportional (P) controller, wirelessly connected to one DSU by means of the CU which acts as the DSU network manager. A computer is used to remotely configure and then start or stop the closed-loop process that is running on the wearable controller without any other communication with the computer.

The closed-loop control relies on 4 sensors: 2 foot pressure insoles that communicate through a Bluetooth 4.0 BLE protocol (FeetMe, France©) with the wearable controller and 2 wired IMU (Bosch© BNO055) that directly provide quaternion estimation. Stimulation is sent via a two-channel DSU to the quadriceps (channel #1) and hamstrings (channel #2) via pairs of surface electrodes located on the skin over the target muscle.

Powered by a commercial USB power bank, a dedicated 3D-printed case (strapped around the waist of the subjects, Figure 6) was designed to host the Raspberry card, the CU acting as a gateway with stimulators' network and the I2C multiplexer used for wired IMU sensors. With up to 8 h of battery life, the FES controller case weighs less than 130 g and measures 9 (length) x 6 (width) x 4 (depth) cm.

**Figure 7:**
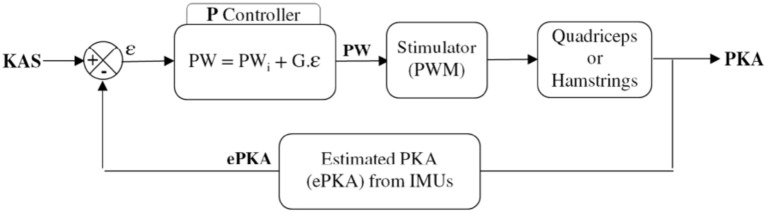
Closed loop control scheme.

Data from IMUs and pressure insoles were periodically acquired and processed online on the controller. Insoles data were used to analyze paretic foot support (PFS) in order to discriminate between stance and swing phases. Stimulation could also be delivered just before initial contact (IC) at the end of the swing phase, in order to anticipate a possible genu recurvatum or crouch gait in stance phase and compensate muscular activation latency. When required and depending on the participant's gait pattern, this “pre-stance” stimulation could be triggered either via an online detection of peak knee flexion or when the sagittal angular speed recorded via the gyroscope crossed zero. In stance phase, stimulation was triggered (*F* = 30 Hz, *I* = 50 mA) either to quadriceps or hamstrings, depending on the paretic knee angle (PKA) estimation relatively to the knee angle set-point (KAS) defined by the practitioner as the optimal flexion during stance phase (around 5°). the P controller adjusted the pulse width (**Figure 9**) depending on the error ϵ between PKA and KAS (Figure 7).

#### 3.2.2. Knee Joint Real-Time Control in Stance Phase

**Figure 8:**
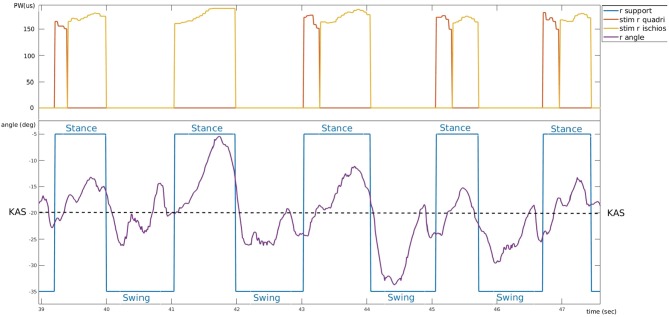
Typical controller behavior. Example of experimental data from participant 3 who suffers a right hemiplegia (trial 4). Bottom: evolution of the right knee angle (purple) around knee angle set-point value (dashed line) and corresponding support phase (blue). Top: electrical stimulation pulse width of right quadriceps (red) and right hamstring (yellow).

**Figure 9:**
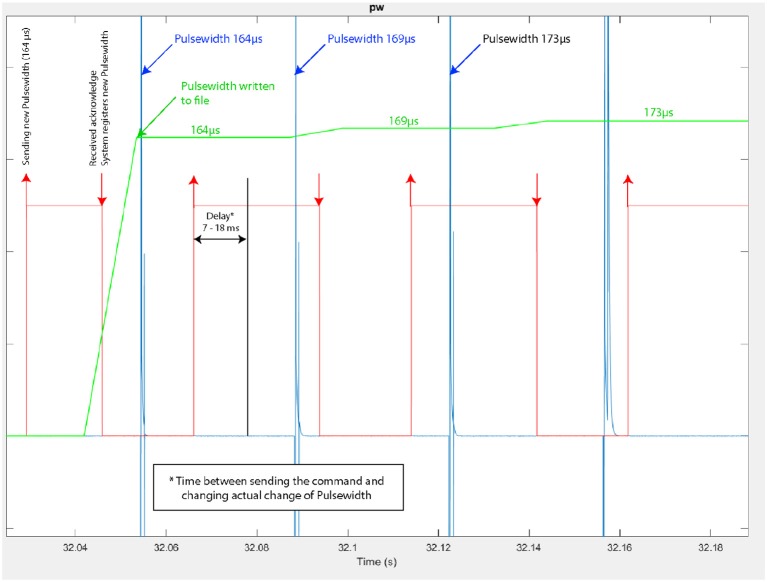
Modulation of the stimulus pulse width during knee joint control (Participant 3–trial 4). In red: time at which a pulse-width modulation request is sent by the controller, indicating also the modulated pulse width value (e.g., 164 μ*s*), in blue: actual pulse-width measured at the stimulator's output, with its current value.

A typical control of the system is shown on Figure 8. 5 gait cycles of participant 3 are plotted. During stance phase (blue), stimulation of hamstrings (yellow) is delivered when PKA (purple line) is higher than the predefined KAS value (dashed line) and stimulation of quadriceps (red) is delivered when PKA is lower than KAS. Stimulation pulse width is adjusted via the P controller depending on the error between PKA and KAS (Figure 9).

The Figure 9 corresponds to a zoom on the time window from 32.04 to 32.18 s of the same trial. We observe more precisely the modulation of the pulse width performed by the closed-loop controller: the pulse-width modulation update at the stimulator output is performed in due time i.e., according to the stimulation frequency.

**Figure 10:**
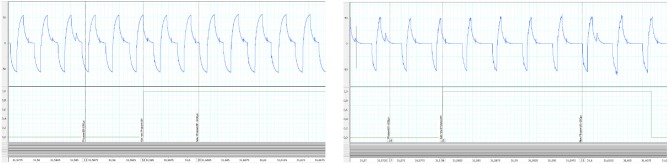
Control latency evaluation from replay (100 Hz) of recorded experimental data: **(Left)** shortest measured latency **(Right)** largest measured latency. In abscissa time is in seconds. Y-Axis are in volt.

We checked the latency of the system from the sensor input at the wearable controller level to the actual output of the channel #1 of the DSU. To do so, acquired data were recorded and then played again with a higher stimulation frequency (*F* increased from 30 to 100 Hz) to allow for a more accurate evaluation of the delay. Results are shown on Figure 10: the maximal measured latency was 18.4 ms and the shortest 7.3 ms. Even in the worst case (due to the fact that the controller must communicate with the CU to control the DSU), this latency is compatible with the dynamics of FES muscle control.

## 4. Discussion and Conclusion

We developed a wireless FES architecture based on a set of distributed stimulation and measurement units managed by a wearable controller. We characterized the performances of the RF network we designed in the proposed distributed FES architecture. The network QoS was observed measuring RTT at every protocol stack layer, as well as LQI and frame loss rate. Time performances reported by RTT measures are highly stable, and medium access is deterministic. This proves that this wireless architecture, with its original STIMAP MAC protocol, is a suitable framework for the deployment of safe closed loop control. The link quality of the 2.4 GHz wireless technology is sensitive to human body attenuation. However, the placement of DUs on the body showed that even in the worst case, frame losses are not critical at all and can be easily avoided. In any case, the CU permanently monitors QoS and both wireless link failure monitoring and safety procedures are implemented on the CU and DUs.

The deterministic and collision free features were demonstrated. Moreover, the accuracy of the timing, linked to the optimization of the STIMAP protocol, shows impressive results with only few μs of delay synchronization error between 2 DSUs simultaneously started through the network.

We assessed the performances of this technology for both open and closed-loop control schemes through a proof-of-concept experimental setup and several applications including the one presented regarding knee joint control in post-stroke patients. A real-time control of the stimulation was also demonstrated using EMG as inputs and the same DSU architecture (Zhan et al., 2018).

The main contribution of this work concerns the design and development of a wireless FES architecture based on dedicated MAC and application layers protocol together with an optimized distribution of the software on DUs. It ensures the flexibility, reliability, and accuracy of this innovative system: adapted to patient / pathology (as regards numbers of DUs), wireless and controllable in closed loop for surface FES with guaranteed timings and safe implementation.

Through this open wearable FES architecture, a scalable hardware solution has been achieved, adaptable to the needs of different FES applications, environments, and pathologies. It is now used by our research team for other applications (Sijobert et al., 2017; Zhan et al., 2018), enabling clinicians to explore novel directions and study new hypotheses.

## Data Availability Statement

All datasets generated and analyzed for this study are included in the article/supplementary material.

## Ethics Statement

The studies involving human participants were reviewed and approved by CPP Nord Ouest I Ethics committee (Trial #017-A03611-52). The patients/participants provided their written informed consent to participate in this study.

## Author Contributions

DA, DG, and MT drafted the manuscript. CA-C and DG supervised the researches. DA and MT Developed the hardware solution. DG had the initial idea of distributed FES concept. CA-C and CF designed the clinical trial. CA-C, CF, and BS participated to the clinical trial.

### Conflict of Interest

MT was employed by the company Vivaltis. The remaining authors declare that the research was conducted in the absence of any commercial or financial relationships that could be construed as a potential conflict of interest.

## Figures and Tables

**Figure A1:**
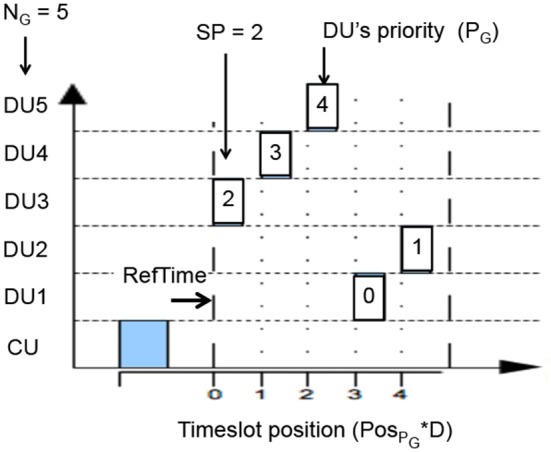
Schematic representation of STIMAP TDMA.

## Appendix

STIMAP is a MAC protocol based on the master/slaves model and dynamic TDMA (details in Andreu et al., 2009). Dynamic TDMA is used dealing with medium access for a group of units (Figure A1). TDMA relies on the allocation of one time-slot of duration *D* to each member of the group. The time-slot duration *D* is the same for all the members of the group. The time-slot duration is dynamically set by the CU (the master) depending on the type of request it sends to the group (the slaves). This means that the CU can define *D* at each group-based communication it induces. For example, a slave needs less time to reply to a test of presence (a simple acknowledgment) than to send back data samples, since the frame sizes are different. So the CU has to adapt *D* to each of these requests. Let's consider a group *G* of *N*_*G*_ members. Each member has a membership number in this group given by *P*_*G*_. The CU sends a request to this group *G*, indicating through its request the time-slot duration *D* and the number of the membership that must start replying, thereafter called starting priority *SP*.

Knowing its membership number in the group, the group size and the starting priority, each member of the group determines itself the position of its time-slot. This position is given by the variable *Pos*_*P*_*G*__, which corresponds to the position of the member *P*_*G*_ in the communication round.

(A1) $$\begin{array}{l} Pos_{P_{G}}=P_{G}-SP+\alpha_{P}\times N_{G} \end{array}$$

where:

- *P*_*G*_ is the membership number (priority from 0 to *N*_*G*_ − 1),
- *SP* is the priority number of the member that must first reply,
- *N*_*G*_ is the *G* group size,
- and α_*p*_ = 1 if *P*_*G*_ < *SP* else α_*p*_ = 0.

And then, from a time point of view, the time-slot position of member *P*_*G*_ is given by:

(A2) $$\begin{array}{l} TimeSlotPosition_{P_{G}}=RefTime_{P_{G}}+Pos_{P_{G}}\times D \end{array}$$

where:

- *D* is the dynamically set time-slot duration,
- and *RefTime*_*P*_*G*__ corresponds to the reference instant of time for member *P*_*G*_. This reference time being defined by:

(A3) $$\begin{array}{l} RefTime_{P_{G}}=\frac{D}{2}-\frac{RTT_{P_{G}}}{2} \end{array}$$
